# Predictors of posttraumatic stress and quality of life in family members of chronically critically ill patients after intensive care

**DOI:** 10.1186/s13613-016-0174-0

**Published:** 2016-07-20

**Authors:** Gloria-Beatrice Wintermann, Kerstin Weidner, Bernhard Strauß, Jenny Rosendahl, Katja Petrowski

**Affiliations:** Department of Psychotherapy and Psychosomatic Medicine, Medizinische Fakultät Carl Gustav Carus, Technische Universität Dresden, Dresden, Germany; Center for Sepsis Control and Care, Jena University Hospital, Friedrich-Schiller University, Jena, Germany; Institute of Psychosocial Medicine and Psychotherapy, Jena University Hospital, Friedrich-Schiller University, Stoystr. 3, 07743 Jena, Germany; Department of Workplace Health Promotion, German Sport University Cologne, Cologne, Germany

**Keywords:** Chronic critical illness (CCI), Intensive care, Posttraumatic stress symptoms, Health-related quality of life (HRQL), Family members, Post-intensive care syndrome (PICS) family, Sepsis

## Abstract

**Background:**

Prolonged mechanical ventilation for acute medical conditions increases the risk of chronic critical illness (CCI). Close family members are confronted with the life-threatening condition of the CCI patients and are prone to develop posttraumatic stress disorder affecting their health-related quality of life (HRQL). Main aim of the present study was to investigate patient- and family-related risk factors for posttraumatic stress and decreased HRQL in family members of CCI patients.

**Methods:**

In a cross-sectional design nested within a prospective longitudinal cohort study, posttraumatic stress symptoms and quality of life were assessed in family members of CCI patients (*n* = 83, aged between 18 and 72 years) up to 6 months after transfer from ICU at acute care hospital to post-acute rehabilitation. Patients admitted a large rehabilitation hospital for ventilator weaning. The Posttraumatic Stress Scale-10 and the Euro-Quality of life-5D-3L were applied in both patients and their family members via telephone interview.

**Results:**

A significant proportion of CCI patients and their family members (14.5 and 15.7 %, respectively) showed clinically relevant scores of posttraumatic stress. Both CCI patients and family members reported poorer HRQL than a normative sample. Factors independently associated with posttraumatic stress in family members were the time following ICU discharge (*β* = .256, 95 % confidence interval .053–.470) and the patients’ diagnosis of PTSD (*β* = .264, 95 % confidence interval .045–.453). Perceived satisfaction with the relationship turned out to be a protective factor for posttraumatic stress in family members of CCI patients (*β* = −.231, 95 % confidence interval −.423 to −.015). Regarding HRQL in family members, patients’ acute posttraumatic stress at ICU (*β* = −.290, 95 % confidence interval −.360 to −.088) and their own posttraumatic stress 3 to 6 months post-transfer (*β* = −.622, 95 % confidence interval −.640 to −.358) turned out to be significant predictors.

**Conclusions:**

Posttraumatic stress and HRQL should be routinely assessed in family members of CCI patients at regular intervals starting early at ICU. Preventive family-centered interventions are needed to improve posttraumatic stress and HRQL in both patients and their family members.

**Electronic supplementary material:**

The online version of this article (doi:10.1186/s13613-016-0174-0) contains supplementary material, which is available to authorized users.

## Background

Treatment on intensive care unit (ICU) for acute medical, surgical, neurologic or cardiac critical illness can go along with secondary medical complications such as severe sepsis with multiorgan failure. Elective placement of tracheotomy and prolonged mechanical ventilation for at least 21 consecutive days put these patients at risk for the development of chronic critical illness (CCI). About 5–10 % of patients requiring prolonged ventilator weaning show an unspecific clinical syndrome of additional key characteristics, e.g., neuromuscular weakness, myopathy, neuropathy, protracted coma, delirium, malnutrition, anasarca, endocrinopathy, inflammatory impairment and intense psychophysiological distress [1, 2]. After discharge from ICU or specialized weaning units, patients still show functional impairments or even ongoing need for ventilator support with an increased risk of death.

The traumatic event of acute critical illness leading to ICU treatment, the heightened 1-year mortality and associated severe physical complications lead to increased caregiving demands and psychological stress in the whole family system of CCI patients [2]. Family members of CCI patients experience a cluster of mental complications [e.g., major depression, complicated grief, acute and posttraumatic stress disorder (ASD/PTSD)] which have been referred to as post-intensive care syndrome family (PICS-F) [3]. Prevalence estimates for clinically relevant posttraumatic stress symptoms in family members of the general ICU population widely range (13–57 %) with a median point prevalence of 21 % [4–7, see systematic reviews: 3, 8, 9]. Highest prevalence rates for posttraumatic stress symptoms in family members of adult general ICU patients have been shown 3 (56 %) [10] and 6 (49 %) [6] months following ICU stay. However, few studies exist assessing the occurrence and severity of posttraumatic stress in family members of CCI patients.

A recent study has shown clinically relevant symptoms of posttraumatic stress in two-third of spouses of CCI patients even an average of 55 months after ICU discharge. Above, higher own or patients’ posttraumatic stress was associated with lower mental and general health-related quality of life (HRQL) in both spouses of CCI patients [11] and in family members of general ICU patients [5]. Furthermore, spouses reported a significantly worse mental HRQL than German normative samples [11].

There is large evidence regarding predictors of posttraumatic stress and impaired HRQL in family members of ICU patients. Characteristics increasing the risk of clinically relevant posttraumatic stress in family members of critically ill patients include female gender, younger age, lower educational level, being an adult child, lifetime mental disorder, higher state or trait anxiety, involvement in decision-making process, bereavement, severity of acute illness and dissatisfaction with information [for an overview see 12]. Family members of younger-aged or critically ill patients were at increased risk of PTSD [13, 14]. Furthermore, the patients’ and their family members’ psychological distress was positively associated up to 6 months following the ICU discharge [e.g., 6]. Regarding the impact of time following ICU discharge on posttraumatic stress in family members, some studies show a decrease in posttraumatic stress, no impact or even an increase with time following ICU discharge [4–6, 11].

Taken together, studies concerning the occurrence of posttraumatic stress in close family members of CCI patients are rare, show heterogeneous results [12], apply various symptom cutoff scores, use either heterogeneous or selective samples of only spouses [11] and consider follow-up periods that are rather short [e.g., 5, 7: 3 months] or quite long [e.g., 11: 55 months]. However, whether results can be replicated in a large, homogeneous and representative sample of both spousal and nonspousal dyads consisting of the CCI patient and their close family members remains to be further elucidated.

Hence, the aims of the present prospective, longitudinal cohort study were the following: first, to investigate whether there were differences in the level of posttraumatic stress and HRQL between CCI patients and their close (spousal or nonspousal) family members. Second aim was to clarify the impact of distinct patient and familymember characteristics as risk factors for the prediction of posttraumatic stress and HRQL in spousal and nonspousal family members of CCI patients following up to 6 months after transfer from ICU at acute care hospital to post-acute rehabilitation.

## Methods

### Setting and procedure

The study was registered at the German Clinical Trials Register (No. DRKS00003386) and approved by the Local Ethics Committee of the Friedrich-Schiller University, Jena, Germany (No 3278-10/11). All patients have signed written informed consent. Family members gave informed consent orally on the telephone.

### Participants and sample size

Criteria for inclusion were a principal diagnosis of critical illness polyneuropathy (CIP; ICD: G62.80) or critical illness myopathy (CIM; ICD: G72.80) with or without sepsis, age between 18 and 72 years, a minimum length of ICU stay of 6 days, sufficient German language skills, informed consent, a negative evaluation of the cognitive test Confusion Assessment Method for the Intensive Care Unit (CAM-ICU) [15, 16] and the presence of a close family member willing to participate in the present study. Patients were excluded from the present study if they were not alert, they were cognitively impaired or they had sensory deficits limiting their ability to communicate. Family members were included if they were at least 18 years of age, gave oral informed consent, showed sufficient German language skills and could be regarded as being closely interrelated with the CCI patient. A close family member was defined as a person most involved in the CCI patient’s treatment and care decisions [7]. Participants were consecutively enrolled. The study was observational with longitudinal and cross-sectional data assessment. Further details describing the study design are reported in [17].

CCI patients were interviewed orally and vis à vis within 4 weeks after transfer from intensive care at acute care hospitals to the ICU in post-acute rehabilitation (t1). Patients were again interviewed via telephone contact 3 (t2) or 6 months (t3) post-transfer. In the following, we refer to the follow-up of t2/t3 as up to 6 months. At t2 or t3, patients were asked whether they had a close family member who would agree to be interviewed. Patients and the patients’ family members were enrolled and interviewed (up to 6 months following transfer to post-acute rehabilitation) if both gave oral informed consent on the telephone. For the present study, a subsample of the already published sample of chronically critically ill patients with data available of their close family members was used [17].

### Measures

Posttraumatic stress was assessed with the German version of the Posttraumatic Symptom Scale (PTSS-10) [18, 19]. The PTSS-10 was applied within a time frame of up to 6 months after transfer from acute care hospital to post-acute rehabilitation in both CCI patients and their close family members. The questionnaire consists of ten items assessing the symptom categories increased arousal, re-experiencing and avoidance according to DSM-III-R criteria [20]. Items are rated on a seven-point Likert scale (1 = never, 7 = always). The total score is received by summing up the scores of all items (range 10–70). A score of more than 35 points is considered as adequate cutoff for clinically relevant PTSD symptomatology [19]. The internal consistency of the PTSS-10 can be regarded as high in the present study (Cronbach’s *α* .87 for patients and .82 for family members). Additionally, the diagnosis of PTSD was ascertained with the Structured Clinical Interview for DSM-IV (SCID) [21] in CCI patients.

Quality of life was assessed with the questionnaire Euro-Quality of Life (EQ-5D-3L) [22] up to 6 months after transfer from acute care hospital to post-acute rehabilitation in CCI patients and their close family members. The EQ-5D-3L measures the HRQL on five dimensions (mobility, self-care, usual activities, pain/discomfort and anxiety/depression) which are evaluated within three severity levels (no problems, some or moderate problems, and extreme problems or unable). Additional, the EQ-5D-3L assesses the current subjective health state via a visual analog scale ranging from 0 (worst health state) to 100 (best health state). A single one-dimensional index value is generated based on a simple sum score according to Hinz et al. [23]. In the present study, Cronbach’s *α* for EQ-5D-3L was .74 for patients and .70 for their family members.

Medical history of the patients was assessed via patient records. Furthermore, the Barthel index was judged by an independent evaluator. Performance in 11 domains comprising activities of daily living (e.g., fecal incontinence, urinary incontinence, help with grooming/toilet use/feeding) is evaluated. Values of Barthel index range between 0 and 100. A higher value is associated with a better mobility and degree of independence from caregivers. Additionally, the early rehabilitation Barthel index was assessed with the seven domains including intensive care supervision, tracheostomy tube management and supervision, intermittent or continuous mechanical ventilation, confusion, behavioral disturbances, severe impairment of communication, and dysphagia, with a minimum value of −325 and a maximum value of 0 [24]. Both Barthel scales were summed up, yielding scores between −325 and 100. Inter-rater reliability is very high (*r* = .95). Test–retest reliability is good as well (*r* = .89) [25].

Acute posttraumatic stress in patients with CCI was measured with the German version of the Acute Stress Disorder Scale (ASDS) [26] within 4 weeks after transfer to post-acute rehabilitation. It consists of 19 items representing symptoms of re-experiencing, avoidance, arousal and dissociation. Patients rated the extent of symptoms on a five-point Likert scale (1 = not at all, 5 = very much). The items are summed up to a total score (range 19–95). In the present study, Cronbach’s *α* was .96. Additionally, the diagnosis of ASD was ascertained with the SCID [21].

### Statistical methods

Normal distribution was tested using the Kolmogorov–Smirnov test. In case of nonnormal distribution, data medians and interquartile ranges are reported. For continuous and normally distributed data, the means and standard deviations were calculated. Categorical variables are reported as absolute and relative frequencies. Bivariate correlational analyses were calculated using point-biserial correlation for a dichotomous and a continuous variable. Wilcoxon’s signed-rank test was used to compare means of outcome variables (HRQL/posttraumatic stress) between patients and family members. McNemar test was conducted in case of dyadic nominal outcome data.

We compared EQ-5D-3L scores of age- and gender-stratified subgroups of our sample with the respective subgroups of a normative German sample [23]. Standardized mean differences (Cohen’s *d*) with 95 % confidence interval (CI) were calculated for these comparisons. Likewise, the patients’ and family members’ PTSS scores were compared to a healthy control group as published by Schüffel et al. [27].

In order to identify risk factors associated with posttraumatic stress (PTSS-10 score) and HRQL (EQ-5D-3L score) in close family members of CCI patients, we first evaluated bivariate correlations (Kendall’s tau, point-biserial correlation). We considered patient-related risk factors (patients’ clinical, acute/chronic psychological and socioeconomic characteristics), family-related risk factors (family members’ socioeconomic, chronic psychological characteristics) and partnership-related risk factors (perceived satisfaction/closeness) (see Additional file 1: Table S1, Additional file 2: Table S2, for a description of univariate correlations see Additional file 3). Second, variables that were correlated (*p* values <.2) with the dependent factor (ASD/PTSD symptomatology) were included in multivariable stepwise regression analyses. PTSS-10 and EQ-5D-3L up to 6 months following transfer to post-acute rehabilitation were considered as outcome variables. Standardized regression coefficients with 95 % CIs were used to quantify the strength of the association.

In the present study, normal distribution of outcome data could not be approved. Analyses were realized using original data. Since multivariate regression analysis is regarded as robust against violations against normal distribution, it was performed nevertheless [28]. Transformation of raw values (empirical *T* values, normal rank-transformed values) only partially revealed normal distribution. A sensitivity analysis without outliers (data>/<2 SD of mean) did not change the main results of our regression model.

We applied a significance level *α* = .05 (two sided). All analyses were performed using SPSS 23 (SPSS Inc., Chicago, IL, USA).

## Results

**Fig. 1:**
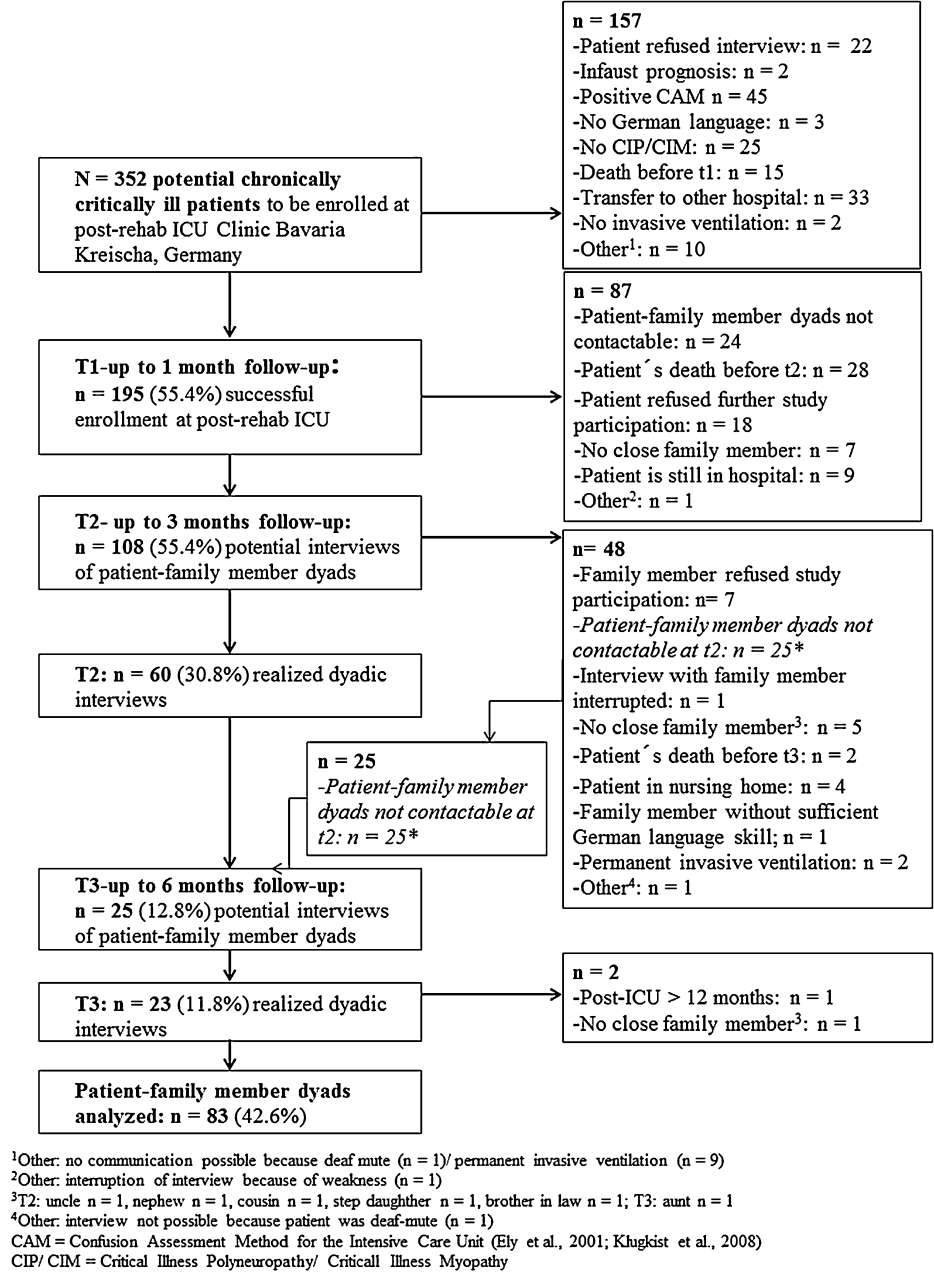
Study flow diagram. *N* = 83 dyads comprising a CCI patients and their close family member were finally analyzed. ^1^Other: no communication possible because deaf-mute (*n* = 1)/permanent invasive ventilation (*n* = 9). ^2^Other: interruption of interview because of weakness (*n* = 1). ^3^T2: uncle *n* = 1, nephew *n* = 1, cousin *n* = 1, step daughter *n* = 1, brother in law *n* = 1; T3: aunt *n* = 1. ^4^Other: interview not possible because patient was deaf-mute (*n* = 1). *CAM* Confusion Assessment Method for the Intensive Care Unit [15, 16]. *CIP/CIM* critical illness polyneuropathy/critical illness myopathy

Of the *N* = 352 potential chronically critically ill patients, *n* = 195 could be successfully enrolled. Of these, *n* = 60 dyadic interviews could be conducted at follow-up about 3 months and further *n* = 23 dyadic interviews about 6 months after transfer from ICU at acute care hospital to post-acute rehabilitation hospital. Finally, data of *n* = 83 patient–family member dyads could be successfully gained up to 6 months following transfer to post-acute rehabilitation hospital (see Fig. 1). In the present study, patients who dropped out of the study revealed significantly more often the respiratory system as site of infection (59.8 %) than patients being followed up (44.6 %). Dropped-out patients showed a significantly lower Barthel index both at discharge from post-acute rehabilitation and at discharge from rehab hospital than patients being followed up (see Additional file 4: Table S3).

Most of the family members were partners (*n* = 59, 71.1 %) comprising husband/wives (*n* = 54, 65.1 %) and cohabitation (*n* = 5, 6.0 %). Other kinds of family members were mother (*n* = 5, 6.0 %), son (*n* = 10, 12.0 %), daughter (*n* = 5, 6.0 %) and sister (*n* = 4, 4.8 %). Family members were significantly younger (median age 59.9) than patients (median age 61.7). Significantly less family members (27.7 %) than patients (73.5 %) were male. Posttraumatic stress was assessed at a median time of 4.8 months following ICU discharge. Patients with CCI stayed at ICU a median time of 66.0 days. Median duration of mechanical ventilation was 50.0 days. An overview of the descriptive characteristics is given in Table 1.

**Table 1:** Descriptive characteristics of the dyads of patients with chronic critical illness and their family members (*n* = 83)

Characteristic	Patients	Family members	*χ* ^2^/*Z*	*p*
Age [years median (IQR)]	61.7 (56.0 to 65.7)	59.9 (48.6 to 64.4)	−2.853	.004*** (*Z*)^a^
Gender [*n* (%)]				
Male	61 (73.5)	23 (27.7)		
Female	22 (26.5)	60 (72.3)	19.014	<.001*** (*χ* ^2^)^b^
Family status [*n* (%)]				
Single	6 (7.2)			
Married/cohabited	63 (75.9)			
Divorced/living apart	8 (9.6)			
Widowed	6 (7.2)			
Characteristics of relationship [median (IQR)]				
Length of partnership^c^		37.0 (25.8 to 43.0)		
Satisfaction with relationship		10.0 (8.0 to 10.0)		
Closeness of relationship		10.0 (9.0 to 10.0)		
Living together in mutual household, yes/no [*n* (%)]		62 (74.7)/21 (25.3)		
Caring for ill patient at the moment, yes/no [*n* (%)]		45 (54.2)/38 (45.8)		
Education [*n* (%)]^d^				
<10 years	26 (31.3)			
≥10 years	54 (65.1)			
ICU stay [days median (IQR)]	66.0 (49.0 to 97.0)			
Mechanical ventilation [days median (IQR)]	50.0 (33.0 to 76.0)			
Sepsis [*n* (%)]				
No sepsis	29 (34.9)			
Sepsis	28 (33.7)			
Severe sepsis or septic shock	26 (31.3)			
Site of infection [*n* (%)]				
Respiratory	37 (44.6)			
Urinary/genitals	8 (9.6)			
Abdominal	8 (9.6)			
Bones/soft tissue	5 (6.0)			
Wound infection	2 (2.4)			
Heart	1 (1.2)			
Multiple	9 (10.8)			
Others^e^	4 (4.8)			
Unknown	4 (4.8)			
Barthel index, median (IQR)				
At admission at post-acute ICU	−200.0 (−225.0 to 140.0)			
At discharge from post-acute rehabilitation	−25.0 (−80.0 to 10.0)			
At discharge from rehab hospital	70.0 (45.0 to 85.0)			
Time following ICU discharge [months median (IQR)]	4.7 (3.8 to 6.4) Min 3.1, Max 9.2	4.8 (3.9–6.5) Min 3.1, Max 9.2	−4.096	<.001*** (*Z*)^a^
Time following mechanical ventilation [months median (IQR)]	3.9 (3.2 to 5.9) Min 1.3, Max 8.5	4.2 (3.3–6.1) Min 1.3, Max 9.4	−4.089	<.001*** (*Z*)^a^

*p ≤ .05

***p ≤ .001

^a^ *Z* value and *p* value from Wilcoxon’s signed-rank test

^b^ *χ* ^2^ (chi-square) value and *p* value from McNemar test

^c^ *n* = 25 missing values

^d^ *n* = 3 missing values

^e^ *n* = 1 brain, *n* = 3 central venous catheter

### Posttraumatic stress and impaired HRQL in patients and their close family members

Patients and their family members did not significantly differ with respect to median posttraumatic stress score as assessed with the PTSS-10 (median, patients: 20.0, family members: 18.0). 14.5 % of patients and 15.7 % of family members showed clinically significant posttraumatic stress symptoms without significant difference between both groups. CCI patients significantly differed regarding PTSS-10 score compared with a healthy control group (*d* = .359, 95 % CI .067–.65). Family members did not significantly differ with respect to posttraumatic stress symptoms compared with a healthy control group (*d* = .245, 95 % CI −.045 to .535). Patients with CCI showed a significantly lower HRQL than their family members. Compared with a healthy German normative sample, HRQL was significantly lower in patients with CCI (*d* = −2.063, 95 % CI −2.291 to −1.834) as well as in their close family members (*d* = −.394, 95 % CI −.614 to −.174) (see Table 2).

**Table 2:** Psychological characteristics of the dyads of patients with CCI and their close family member (*n* = 83)

Characteristic	Patients	Family members	*Z*/*χ* ^2^	*p*
Posttraumatic Stress Scale				
Median (IQR)^a^	20.0 (14.0–29.0)	18.0 (14.0–28.0)	−1.029	.303 (*Z*)^b^
Posttraumatic Stress Scale^a^ ≥ 35 (%)	12 (14.5)	13 (15.7)	.000	1.000 (*χ* ^2^)^c^
Quality of life^d^				
Median (IQR)	70.0 (50.0–80.0)	90.0 (80.0–100.0)	−6.529	<.001*** (*Z*)^b^
History of traumatic life experiences				
Yes [*n* (%)]^e^	24 (28.9)^e^	46 (55.4)	5.281	.022* (*χ* ^2^)^c^

*p ≤ .05

***p ≤ .001

^a^PTSS-10 [19]; ^b^ *Z* value and *p* value from Wilcoxon’s signed-rank test; ^c^ *χ* ^2^ (chi-square) value and *p* value from McNemar test; ^d^ EQ-5D-3L formula according to Hinz et al. [23]; ^e^ *n* = 14 (16.9 %) missing values

There was a significant association between patients’ and family members’ posttraumatic stress (PTSS-10, *τ* = .167, *p* = .030). Concerning HRQL, no dyadic association could be found (EQ-5D-3L, *τ* = .108, *p* = .216). Both groups, family members and CCI patients, showed a significant negative association between their PTSS-10 scores and their EQ-5D-3L scores (actor effect) (PTSS-10 × EQ-5D-3L, family members: *τ* = −.367, *p* < .001; patients: *τ* = −.384, *p* < .001). Patients and family members with higher posttraumatic stress reported lower HRQL. Regarding the effect of posttraumatic stress on their respective partners’ HRQL (partner effect), there was no significant impact in neither of the two groups (PTSS-10 × EQ-5L, family members: *τ* = −.135, *p* = .095; patients: *τ* = −.068, *p* = .413).

### Predictors of posttraumatic stress in family members of CCI patients

Up to 6 months following discharge from ICU of CCI patients, the time following ICU discharge (*β* = .262, 95 % CI .061–.476) and a diagnosis of PTSD up to 6 months following ICU discharge in CCI patients (*β* = .254, 95 % CI .089–1.102) could be identified as significant patient-related characteristics predictive of posttraumatic stress in family members. With respect to family-related characteristics, the perceived satisfaction with the relationship could be identified (*β* = −.229, 95 % CI −.425 to −.013). The model explained an adjusted total variance of 17.9 % [*R*^2^ = .18, *F*(3, 77) = 6.828, *p* < .001] (Table 3).

**Table 3:** Multiple stepwise regression analysis with patient and family member characteristics as regressors and posttraumatic stress in family member of CCI patients as dependent variable *N* = (83)

Predictors of posttraumatic stress in family members of CCI patients	*β*	95 % CI	*T*	*p*
Characteristics of patients with CCI				
Diagnosis of PTSD, 3–6 months following ICU	.254	.089 to 1.102	2.342	.022*
Time following ICU	.262	.061 to .476	2.571	.012*
Characteristics of close family members				
Satisfaction with relationship	−.229	−.425 to −.013	−2.122	.037*

*p ≤ .05

### Predictors of HRQL in family members of CCI patients

Within 3 to 6 months following discharge from ICU of the CCI patient, their own PTSS-10 score (*β* = −.622, 95 % CI −.640 to −.358) and the acute posttraumatic stress at ICU as assessed with the ASDS in CCI patients (*β* = −.290, 95 % CI −.360 to −.088) could be identified as significant predictors of HRQL in close family members. The model explained an adjusted total variance of 47.4 % [*R*^2^ = .47, *F*(2, 66) = 31.583, *p* < .001] (see Additional file 5: Table S4).

## Discussion

The primary aim of the present study was the investigation of the level of posttraumatic stress and HRQL in close family members of CCI patients with or without sepsis following 3 to 6 months after ICU discharge. The secondary aim was on the examination of dyadic associations between posttraumatic stress and HRQL between patients and their family members. Third, we intended to specify patient- and family-related predictors of posttraumatic stress and HRQL in close family members of CCI patients.

Clinically relevant symptomatology of PTSD was a problem for both patients and their close family members. Nearly every sixth close family member and every seventh patient surviving CCI displays symptoms of clinically significant posttraumatic stress up to 6 months following ICU. Both groups showed a significantly reduced HRQL compared with a German normative sample [23]. CCI patients displayed an EQ-5D-3L score approximately two standard deviations below the mean of a representative healthy German sample. Regarding the effect of posttraumatic stress on HRQL, actor effects but no partner effects could be elucidated. Significant patient-related predictors for posttraumatic stress in family members of CCI patients 3 to 6 months following ICU discharge were the time following ICU and a diagnosis of post-ICU PTSD. The perceived satisfaction with the relationship was associated with a decreased PTSS-10 score. Regarding HRQL in close family members of patients with CCI, a higher posttraumatic stress at post-rehab ICU in patients and their own posttraumatic stress were significant predictors.

Clinically significant posttraumatic stress symptoms are among the most common psychological long-term sequelae in intensive care unit survivors with a median point prevalence of 19 % [e.g., 29, 30]. Also family members of critically ill patients are at risk of posttraumatic stress disorder both during and after the ICU stay as second-order patients. The critical illness and admission to the ICU are life-threatening events putting the whole family on a severe emotional crisis with profound changes in family roles and responsibilities [31]. Moreover, caregiver of CCI patients experiences distress which presumably arises from patients’ problem behaviors such as negative emotions or pain. Above, family members perceive restrictions in social life and personal recreation [32]. The rate of clinically relevant posttraumatic stress symptoms in our study was 15.7 % for close family members and 14.5 % for CCI patients. This frequency is a bit higher than the rate found in a sample of older adults seeking medical services in primary health clinics (11.1 %) [33] but in line with a former study showing a prevalence rate of 14.0 % for family members of patients dying at the ICU [13]. Jubran et al. [34] also found a lower prevalence rate of 12.0 % for diagnosed PTSD in patients surviving weaning from prolonged mechanical ventilation. Lower prevalence rates in the present study contradict higher prevalence rates in former studies [e.g., 4–6, 11]. This might be justified by differences in the applied methods and cutoffs to assess posttraumatic stress. For instance, the lowest prevalence rates of PTSD have been found in studies using self-report symptom checklists as item mapping approach based on DSM-IV criteria for PTSD [13, 35]. Moreover, higher prevalence rates were more common in homogeneous sample of spouses than other kinship relations [e.g., 11, 14]. In contrast, other studies also comprising nonspousal family members, patient’s next of kins or designated power of attorney for healthcare even found higher rates of posttraumatic stress [4–6, 36]. Thus, the specific characteristics of the respective study samples might have played a major role. Former studies could show highest prevalence rates in family members whose relative died in the ICU and who shared in end-of-life decisions [5, 12, 37]. In the present study, only CCI patients who survived the follow-up period were included. Moreover, former studies could approve younger age as significant predictor of post-ICU posttraumatic stress. Following, lower prevalence rates in the present study might be ascribed to older age since nearly 50 % of the family members were aged 60 years or older [37, 38].

The present study could confirm a negative association between posttraumatic stress and HRQL in both CCI patients and their close family member after intensive care [11]. Our results are also in line with the study by Chung et al. [39] showing a significant actor effect for depressive and anxiety symptoms on quality of life in both patients with heart failure and their spouses. The increased and ongoing need for care during the post-acute recovery phase demands close interactions between patients and their family members. This launches a contagious process of emotional transmission leading to the induction of feelings of empathy in the members of the dyad. Consequently, a patient’s HRQL can be affected not only by his/her own mental health status (actor effect) but also by their respective family member’s one (partner effect) [11]. In the present study, no partner effects could be shown. Also former studies could not consistently reveal partner effects. Chung et al. [39] could only show partner effects for spouses of patients with heart failure. Those spouses with high depressive/anxiety symptoms negatively impacted the quality of life in the respective patients. Rosendahl et al. [11] could only show partner effects for posttraumatic stress symptoms of patients surviving severe sepsis on their spouses’ mental HRQL. Another study in cancer patients showed a transmission of emotional stress only from male patients to their female partners [40]. A possible explanation of the missing partner effects for the present study might be the inclusion of a heterogeneous sample of both spouses and other family members (sister, daughter, son, etc.). Moreover, the EQ-5D-3L is not sufficiently sensitive for the assessment of the mental component of HRQL or a differential representation of social and emotional role functioning which might be disturbed in family members of CCI patients.

Another influential factor displays the time frame for the assessment of posttraumatic stress symptoms in CCI patients and their close family members. Former studies have shown the highest prevalence rates for posttraumatic stress symptoms associated with a moderate to major risk of PTSD 3 months [e.g., 5, 7, 10] and 6 months [e.g., 6] following ICU discharge. Findings even suggest that intensity of PTSD symptoms declines in the follow-up of the ICU experience [7] and there is a considerable rate showing recovery with decreasing PTSD symptoms post-ICU [41]. However, in the present study family members’ posttraumatic stress could be significantly predicted by the time following ICU discharge. Since illness-associated morbidity in this particular sample of CCI patients persists for years, it is well known that several months after ICU discharge the physical and emotional burden of disease endures in these patients and their family [2]. CCI patients exhibit an increased hospital lethality of about 50 % and a 1-year survival rate of only 25 % [42, 43]. Following, the patient’s hospital discharge does not resolve psychological stress for the family system. Hospital readmissions and the development of new or worsening impairments in physical, cognitive or mental health status [30] expose the family members to a chronic state of psychological arousal. Thus, vulnerability for clinically relevant symptoms of posttraumatic stress is increased even in the follow-up after ICU discharge. This finding is further corroborated by a considerable rate of delayed onset or chronic/persistent course of PTSD during longer follow-up periods in the aftermath of ICU stay [41, 44].

Another major finding of the present study is the prediction of HRQL in family members of CCI patients by the patients’ acute posttraumatic stress at ICU. To the best of our knowledge, this is the first study prospectively assessing patient-related characteristics and associating them with HRQL in close family members of CCI patients. The intensity of acute posttraumatic stress experienced by CCI patients within 1 month following transfer from acute care hospital mirrors the severity of the traumatic event the patients were exposed to and the acute psychological reactions in the ICU (e.g., agitation, extreme fear, early intrusive memories, delirious symptoms, mood disturbance) putting those patients at increased risk of persistent or delayed PTSD in the follow-up [45]. Following, the more distressed and emotionally aroused patients are during ICU stay the more they impact their close family members’ emotional as well as physical well-being. This could be shown in the present study by a, although small, but significantly positive correlation between posttraumatic stress in CCI patients and their close family member [see also 46]. Furthermore and up to a certain extent, the present finding could be corroborated by a study showing an association between psychological distress in patients 2 weeks after ICU discharge and PTSD scores in their close family members at 6-month follow-up [6]. Another predictor of family members’ posttraumatic stress was the satisfaction with the relationship. This family-related factor turned out to be protective and might moderate the relationship between emotional distress and quality of life [39].

We could not find a significant effect of the severity of illness, the length of ICU stay, family member’s age and gender. This is in line with existing findings [e.g., 11, 47] but on the other hand contradicts results showing a significant impact of caregivers’ sex and length of ICU stay [48]. Jones et al. [6] did not find any association between the family members’ posttraumatic stress and demographic characteristics related to the patient’s ICU stay which confirms our findings. Above, sepsis has not been consistently elucidated as significant risk factor for post-ICU psychological morbidity in patients and their family members [17, 49–51]. The present findings confirm lower HRQL in women and participants of older age. This has been already shown in former studies [e.g., 23].

For the present study, some methodological shortcomings should be addressed. First, for the assessment of posttraumatic stress disorder a self-report measure (PTSS-10) instead of a clinician administered structured diagnostic interview was applied. The PTSS-10 is based on subjectively reported symptom intensities. Thus, a potential overestimation of the frequency of posttraumatic stress might have occurred. Above, the PTSS-10 has some limitations since it is based on DSM-III criteria, items on numbing or flashbacks are missing and only one item assesses avoidance. Future study should rather use the PTSS-14 [52] as extended version for a more accurate assessment of posttraumatic symptomatology. Second, the recruitment of family members by phone contact might have been prone to socially desirable answers and bias. Third, since the nature of the present study design is, although nested within a prospective study, rather observational and cross-sectional, no causal interference is possible. There is no information about posttraumatic stress in close family members of CCI patients at different time points following ICU discharge. Also, there is no information concerning the number of family members demanding psychological support or having been treated for their PTSD. Fourth, longitudinal and multicenter trials are needed and should focus on the long-term course of physical and mental health in patient–family member dyads after critical illness taking into account salutogenetic aspects of dyadic dynamics. Above, future studies should address the effectiveness of interventions (e.g., realization of the recommendations suggested in the pain, agitation and delirium clinical practice guidelines, establishment of Critical Care Recovery Centers (CCRC) [53]; family-centered interventions improve dyadic coping strategies [8]) in preventing post-intensive care syndrome, enhancing post-critical illness rehabilitation and improving quality of life in patients and their families after the ICU experience [3]. Additionally, future research should address the impact the family members’ PTSD might have on the dyadic relationship and the CCI patients’ recovery process. Moreover, the patients’ family history of PTSD should be more closely focused on in order to identify genetic or sociological risk factors allowing targeted interventions in a risk population. With the aim to launch preventive interventions for effective care of clinically relevant PTSD in close family members of CCI patients, the early ascertainment of patient- as well as family-related risk factors and diagnostic markers for posttraumatic stress in close family members of CCI patients is highly warranted.

## Conclusions

There is a considerable rate of clinically relevant posttraumatic stress symptoms and significantly diminished HRQL in close family members of CCI patients following ICU stay. Close family members of CCI patients display second-order patients which also suffer from impaired mental and physical long-term sequelae following CCI and intensive care. We suggest a routine assessment of patient- and family-related risk factors while the chronically critically ill patient is in the ICU and up to 6 months following discharge from ICU at acute care hospital. Posttraumatic stress and quality of life should be ascertained at regular intervals starting at ICU admission and being followed up at least 6 months post-ICU.

## Additional files

10.1186/s13613-016-0174-0 Unadjusted univariate analyses to determine the association between risk factors (patient-related clinical, socioeconomic, acute psychological and chronic psychological factors; family-related socioeconomic, chronic psychological factors, characteristics of the partnership) and post-ICU posttraumatic stress as assessed with the PTSS-10 (Posttraumatic Stress Scale, Raphael et al., 1989) in close family members of patients with chronically critical illness up to six months after discharge from ICU at acute care hospital (sample: n = 83).
10.1186/s13613-016-0174-0 Unadjusted univariate analyses to determine the association between risk factors (patient-related clinical, socioeconomic, acute psychological and chronic psychological factors; family -related socioeconomic, acute psychological and chronic psychological factors, characteristics of the partnership) and post-ICU health-related quality of life as assessed with the EQ-5D-3L (Rabin & de Charro, 2001) in close relatives of patients with chronically critical illness up to six months after discharge from ICU (sample: n = 83).
10.1186/s13613-016-0174-0 Description of univariate correlations.
10.1186/s13613-016-0174-0 Socio-demographic and clinical characteristics of the patients being followed up (n = 83) and drop outs (n = 112).
10.1186/s13613-016-0174-0 Multiple stepwise regression analysis with patient and family member characteristics as regressors and health-related quality of life in family member of CCI patients as dependent variable N = (83).

## Abbreviations

ASDS: Acute Stress Disorder Scale
ASD: acute stress disorder
CCI: chronic critical illness
CAM-ICU: Confusion Assessment Method for the Intensive Care Unit
CI: confidence interval
EQ-5D-3L: Euro-Quality of Life
ICU: intensive care unit
PICS-F: post-intensive care syndrome family
PTSS-10: Posttraumatic Symptom Scale
PTSD: posttraumatic stress disorder
SCID: Structured Clinical Interview for DSM-IV

## References

[1] Marchioni A, Fantini R, Antenora F, Clini E, Fabbri L (2015). Chronic critical illness: the price of survival. Eur J Clin Invest.

[2] Nelson JE, Cox CE, Hope AA, Carson SS (2010). Chronic critical illness. Am J Respir Crit Care Med.

[3] Davidson JE, Jones C, Bienvenu OJ (2012). Family response to critical illness: postintensive care syndrome-family. Crit Care Med.

[4] Anderson WG, Arnold RM, Angus DC, Bryce CL (2008). Posttraumatic stress and complicated grief in family members of patients in the intensive care unit. J Gen Intern Med.

[5] Azoulay E, Pochard F, Kentish-Barnes N, Chevret S, Aboab J, Adrie C (2005). Risk of post-traumatic stress symptoms in family members of intensive care unit patients. Am J Respir Crit Care Med.

[6] Jones C, Skirrow P, Griffiths RD, Humphris G, Ingleby S, Eddleston J (2004). Post-traumatic stress disorder-related symptoms in relatives of patients following intensive care. Intensive Care Med.

[7] McAdam JL, Fontaine DK, White DB, Dracup KA, Puntillo KA (2012). Psychological symptoms of family members of high-risk intensive care unit patients. Am J Crit Care.

[8] Petrinec AB, Mazanec PM, Burant CJ, Hoffer A, Daly B (2015). Coping strategies and posttraumatic stress symptoms in post-ICU family decision makers. Crit Care Med.

[9] van Beusekom I, Bakhshi-Raiez F, de Keizer NF, Dongelmans DA, van der Schaaf M (2016). Reported burden on informal caregivers of ICU survivors: a literature review. Crit Care.

[10] Lautrette A, Darmon M, Megarbane B, Joly LM, Chevret S, Adrie C (2007). A communication strategy and brochure for relatives of patients dying in the ICU. N Engl J Med.

[11] Rosendahl J, Brunkhorst FM, Jaenichen D, Strauss B (2013). Physical and mental health in patients and spouses after intensive care of severe sepsis: a dyadic perspective on long-term sequelae testing the Actor–Partner Interdependence Model. Crit Care Med.

[12] Hickman RL, Douglas SL (2010). Impact of chronic critical illness on the psychological outcomes of family members. AACN Adv Crit Care.

[13] Gries CJ, Engelberg RA, Kross EK, Zatzick D, Nielsen EL, Downey L (2010). Predictors of symptoms of posttraumatic stress and depression in family members after patient death in the ICU. Chest.

[14] Siegel MD, Hayes E, Vanderwerker LC, Loseth DB, Prigerson HG (2008). Psychiatric illness in the next of kin of patients who die in the intensive care unit. Crit Care Med.

[15] Ely EW, Inouye SK, Bernard GR, Gordon S, Francis J, May L (2001). Delirium in mechanically ventilated patients: validity and reliability of the Confusion Assessment Method for the Intensive Care Unit (CAM-ICU). JAMA.

[16] Klugkist M, Sedemund-Adib B, Schmidtke C, Schmucker P, Sievers HH, Hüppe M (2008). Confusion Assessment Method for the Intensive Care Unit (CAM-ICU): diagnosis of postoperative delirium in cardiac surgery. Anaesthesist.

[17] Wintermann GB, Brunkhorst FM, Petrowski K, Strauss B, Oehmichen F, Pohl M (2015). Stress disorders following prolonged critical illness in survivors of severe sepsis. Crit Care Med.

[18] Raphael B, Lundin T, Weisaeth L (1989). A research method for the study of psychological and psychiatric aspects of disaster. Acta Psychiatr Scand Suppl.

[19] Stoll C, Kapfhammer HP, Rothenhausler HB, Haller M, Briegel J, Schmidt M (1999). Sensitivity and specificity of a screening test to document traumatic experiences and to diagnose post-traumatic stress disorder in ARDS patients after intensive care treatment. Intensive Care Med.

[20] American Psychiatric Association (1980). Diagnostic and statistical manual of mental disorders.

[21] Wittchen HU, Wunderlich U, Gruschwitz S, Zaudig M (1997). Structured Clinical Interview for DSM disorders (SCID).

[22] Rabin R, de Charro F (2001). EQ-5D: a measure of health status from the EuroQol Group. Ann Med.

[23] Hinz A, Brahler E, Schwarz R, Schumacher J, Stirn A (2005). How useful is the calculation of total scores for questionnaires concerning health related quality of life?. Psychother Psychosom Med Psychol.

[24] Rollnik JD (2011). The Early Rehabilitation Barthel Index (ERBI). Rehabilitation (Stuttg).

[25] O’Sullivan SB, Schmitz TJ (2006). Physical rehabilitation.

[26] Helfricht S, Landolt MA, Moergeli H, Hepp U, Wegener D, Schnyder U (2009). Psychometric evaluation and validation of the German version of the Acute Stress Disorder Scale across two distinct trauma populations. J Trauma Stress.

[27] Schüffel W, Schade B, Schunk T. A brief inventory to investigate stress reactions: Posttraumatic Symptom Scale, 10 Items (PTSS-10) by Raphael, Lundin & Weisaeth. Marburg, Paper. http://psydok.sulb.uni-saarland.de/volltexte/2004/437/pdf/artikel.pdf. Accessed 17 Mar 2016.

[28] van Belle G (2002). Statistical rules of thumb.

[29] Davydow DS, Gifford JM, Desai SV, Needham DM, Bienvenu OJ (2008). Posttraumatic stress disorder in general intensive care unit survivors: a systematic review. Gen Hosp Psychiatry.

[30] Needham DM, Davidson J, Cohen H, Hopkins RO, Weinert C, Wunsch H (2012). Improving long-term outcomes after discharge from intensive care unit: report from a stakeholders’ conference. Crit Care Med.

[31] Johnson SK, Craft M, Titler M, Halm M, Kleiber C, Montgomery LA (1995). Perceived changes in adult family members’ roles and responsibilities during critical illness. Image J Nurs Sch.

[32] Choi J, Donahoe MP, Zullo TG, Hoffman LA (2011). Caregivers of the chronically critically ill after discharge from the intensive care unit: six months’ experience. Am J Crit Care.

[33] Preville M, Lamoureux-Lamarche C, Vasiliadis HM, Grenier S, Potvin O, Quesnel L (2014). The 6-month prevalence of posttraumatic stress syndrome (PTSS) among older adults: validity and reliability of the PTSS scale. Can J Psychiatry.

[34] Jubran A, Lawm G, Duffner LA, Collins EG, Lanuza DM, Hoffman LA (2010). Post-traumatic stress disorder after weaning from prolonged mechanical ventilation. Intensive Care Med.

[35] Jackson JC, Pandharipande PP, Girard TD, Brummel NE, Thompson JL, Hughes CG (2014). Depression, post-traumatic stress disorder, and functional disability in survivors of critical illness in the BRAIN-ICU study: a longitudinal cohort study. Lancet Respir Med.

[36] Sundararajan K, Martin M, Rajagopala S, Chapman MJ (2014). Posttraumatic stress disorder in close Relatives of Intensive Care unit patients’ Evaluation (PRICE) study. Aust Crit Care.

[37] Fumis RR, Ranzani OT, Martins PS, Schettino G (2015). Emotional disorders in pairs of patients and their family members during and after ICU stay. PLoS ONE.

[38] Girard TD, Shintani AK, Jackson JC, Gordon SM, Pun BT, Henderson MS (2007). Risk factors for post-traumatic stress disorder symptoms following critical illness requiring mechanical ventilation: a prospective cohort study. Crit Care.

[39] Chung ML, Moser DK, Lennie TA, Rayens MK (2009). The effects of depressive symptoms and anxiety on quality of life in patients with heart failure and their spouses: testing dyadic dynamics using Actor–Partner Interdependence Model. J Psychosom Res.

[40] Moser MT, Kunzler A, Nussbeck F, Bargetzi M, Znoj HJ (2013). Higher emotional distress in female partners of cancer patients: prevalence and patient-partner interdependencies in a 3-year cohort. Psychooncology.

[41] Myhren H, Ekeberg O, Toien K, Karlsson S, Stokland O (2010). Posttraumatic stress, anxiety and depression symptoms in patients during the first year post intensive care unit discharge. Crit Care.

[42] Angus DC, Carlet J (2003). Surviving intensive care: a report from the 2002 Brussels Roundtable. Intensive Care Med.

[43] Marik PE (2010). Handbook of evidence-based critical care.

[44] Schelling G, Briegel J, Roozendaal B, Stoll C, Rothenhäusler HB, Kapfhammer HP (2001). The effect of stress doses of hydrocortisone during septic shock on posttraumatic stress disorder in survivors. Biol Psychiatry.

[45] Wade DM, Howell DC, Weinman JA, Hardy RJ, Mythen MG, Brewin CR (2012). Investigating risk factors for psychological morbidity three months after intensive care: a prospective cohort study. Crit Care.

[46] Hagedoorn M, Sanderman R, Bolks HN, Tuinstra J, Coyne JC (2008). Distress in couples coping with cancer: a meta-analysis and critical review of role and gender effects. Psychol Bull.

[47] Paparrigopoulos T, Melissaki A, Efthymiou A, Tsekou H, Vadala C, Kribeni G (2006). Short-term psychological impact on family members of intensive care unit patients. J Psychosom Res.

[48] Andresen M, Guic E, Orellana A, Diaz MJ, Castro R (2015). Posttraumatic stress disorder symptoms in close relatives of intensive care unit patients: prevalence data resemble that of earthquake survivors in Chile. J Crit Care.

[49] Bienvenu OJ, Gellar J, Althouse BM, Colantuoni E, Sricharoenchai T, Mendez-Tellez PA (2013). Post-traumatic stress disorder symptoms after acute lung injury: a 2-year prospective longitudinal study. Psychol Med.

[50] Boer KR, Mahler CW, Unlu C, Lamme B, Vroom MB, Sprangers MA (2007). Long-term prevalence of post-traumatic stress disorder symptoms in patients after secondary peritonitis. Crit Care.

[51] Davydow DS, Hough CL, Langa KM, Iwashyna TJ (2012). Depressive symptoms in spouses of older patients with severe sepsis. Crit Care Med.

[52] Twigg E, Humphris G, Jones C, Bramwell R, Griffiths RD (2008). Use of a screening questionnaire for post-traumatic stress disorder (PTSD) on a sample of UK ICU patients. Acta Anaesthesiol Scand.

[53] Khan BA, Lasiter S, Boustani MA (2015). CE: critical care recovery center: an innovative collaborative care model for ICU survivors. Am J Nurs.

