# Supplementary material for: Predictors of posttraumatic stress and quality of life in family members of chronically critically ill patients after intensive care
Source: Ann Intensive Care. 2016 Jul 20;6:69. doi: 10.1186/s13613-016-0174-0 (PMC4954797; doi:10.1186/s13613-016-0174-0)
Supplement: Supplementary file 1 — 10.1186/s13613-016-0174-0 Unadjusted univariate analyses to determine the association between risk factors (patient-related clinical, socioeconomic, acute psychological and chronic psychological factors; family-related socioeconomic, chronic psychological factors, characteristics of the partnership) and post-ICU posttraumatic stress as assessed with the PTSS-10 (Posttraumatic Stress Scale, Raphael et al., 1989) in close family members of patients with chronically critical illness up to six months after discharge from ICU at acute care hospital (sample: n = 83). [file 13613_2016_174_MOESM1_ESM.docx]

**Table S1:** Unadjusted univariate analyses to determine the association between risk factors (patient-related clinical, socioeconomic, acute psychological and chronic psychological factors; family-related socioeconomic, chronic psychological factors, characteristics of the partnership) and post-ICU posttraumatic stress as assessed with the PTSS-10 (Posttraumatic Stress Scale, Raphael et al., 1989) in close family members of patients with chronically critical illness up to six months after discharge from ICU at acute care hospital (sample: n = 83).

|  | | **PTSS-10 score in close family members up to six months post-ICU** | | ***p*** |
| --- | --- | --- | --- | --- |
| **Characteristics of patients with CCI** | | | | |
| **Clinical characteristics** | | | | |
| total duration of ICU stay | | τ = .028^1^ | | p = .711 |
| total duration of ventilation | | τ = -.046^1^ | | p = .544 |
| Barthel index at admission at post-rehab hospital | | τ = -.075^1^ | | p = .349 |
| Barthel index at discharge from post-rehab hospital | | τ = -.003^1^ | | p = .972 |
| Barthel index at discharge from rehab hospital | | τ = .062^1^ | | p = .429 |
| diagnosis of sepsis | | r = .022^2^ | | p = .842 |
| severity of sepsis | | τ = .090^1^ | | p = .299 |
| Time following ICU discharge | | τ = .165^1^ | | **p = .030*** |
| Time following mechanical ventilation^3^ | | τ = .188^1^ | | **p = .014*** |
| **Socioeconomic characteristics** | | | | |
| age | | τ = -.115^1^ | | p = .130 |
| sex | | r = -.183^2^ | | p = .098 |
| family status (living in partnership vs. not living in partnership) | | r = -.042^2^ | | p = .703 |
| educational level (≥ 10 years at school vs. < 10 years at school) | | r = -.062^2,4^ | | p = .584 |
| **Acute psychological characteristics** | | | | |
| perceived helplessness at ICU | | τ = -.041^1,4^ | | p = .618 |
| perceived fear of dying at ICU | | τ = .022^1,4^ | | p = .796 |
| ASDS^5^ score at ICU | | τ = .020^1,6^ | | p = .825 |
| diagnosis of ASD^7^ at ICU (SCID-I ^8^) | | r = .078^2,6^ | | p = .488 |
| **Chronic psychological health conditions** | | | | |
| number of traumatic memories from ICU (up to six months following ICU) | | τ = .051^1,9^ | | p = .541 |
| diagnosis (SCID-I^7^) of PTSD (up to six months following ICU) | | r = .303^2,9^ | | **p = .006**** |
| PTSS-10 score (up to six months following ICU)^10^ | | τ = .167 | | **p = .030*** |
| previous psychiatric history^11^ | | r = .155^2^ | | p = .163 |
| history of alcohol consumption | | r = .134^2^ | | p = .226 |
| history of anxiety disorder | | r = .115^2^ | | p = .301 |
| history of affective disorder | | r = .212^2^ | | p = .055 |
| history of previous traumatic life experiences | | r = -.029^2,12^ | | p = .812 |
| **Characteristics of close family members** | | | | |
| **Socioeconomic characteristics** | | | | |
| age^13^ | | τ = -.087^1^ | | p = .250 |
| sex | r = .190^2^ | | p = .085 | |
| **Chronic psychological health conditions** | | | | |
| history of previous traumatic life experiences | r = .022^2^ | | p = .841 | |
| **Characteristics of relationship** | | | | |
| perceived satisfaction with relationship | | τ = -.201^1,9^ | | **p = .019*** |
| perceived closeness in relationship | | τ = .042^1^ | | p = .630 |

^1^ Kendall´s τ; ^2^point-biserial correlation; ^3^Time following ICU discharge and Time following mechanical ventilation are intercorrelated (τ = .605, p < .001). For parsimony of the final model and in accordance with the present study design we chose time following ICU discharge from acute care hospital as predictor for the final model.^4^n = 3 missing values; ^5^ASDS (Acute Stress Disorder Scale, Helfricht et al., 2009); ^6^n = 2 missing values; ^7^ASD (Acute Stress Disorder); ^8^SCID (Structured Clinical Interview DSM-IV, Wittchen et al., 1997); ^9^n = 1 missing value; ^10^PTSS-10 score (up to six months following ICU) and diagnosis of PTSD (up to six months following ICU) were highly intercorrelated (r = .403, p < .001). For parsimony of the final model only diagnosis of PTSD was included in the final model. ^11^History of previous psychiatry history and history of affective disorder were highly correlated (contingency coefficient r = .385, p < .001). For parsimony of the final model only history of affective disorder was included in the final model. ^12^n = 14 missing values; ^13^Age of patient and age of close family members were highly intercorrelated (τ = .992, p < .001). For parsimony of the final model only age of close family members was included in the final model. * p<.05
