# Supplementary material for: Predictors of posttraumatic stress and quality of life in family members of chronically critically ill patients after intensive care
Source: Ann Intensive Care. 2016 Jul 20;6:69. doi: 10.1186/s13613-016-0174-0 (PMC4954797; doi:10.1186/s13613-016-0174-0)
Supplement: Supplementary file 2 — 10.1186/s13613-016-0174-0 Unadjusted univariate analyses to determine the association between risk factors (patient-related clinical, socioeconomic, acute psychological and chronic psychological factors; family -related socioeconomic, acute psychological and chronic psychological factors, characteristics of the partnership) and post-ICU health-related quality of life as assessed with the EQ-5D-3L (Rabin & de Charro, 2001) in close relatives of patients with chronically critical illness up to six months after discharge from ICU (sample: n = 83). [file 13613_2016_174_MOESM2_ESM.docx]

**Table S2:** Unadjusted univariate analyses to determine the association between risk factors (patient-related clinical, socioeconomic, acute psychological and chronic psychological factors; family -related socioeconomic, acute psychological and chronic psychological factors, characteristics of the partnership) and post-ICU health-related quality of life as assessed with the EQ-5D-3L (Rabin & de Charro, 2001) in close relatives of patients with chronically critical illness up to six months after discharge from ICU (sample: n = 83).

|  | | **EQ-5D-3L score in close family members up to six months post-ICU** | | ***p*** |
| --- | --- | --- | --- | --- |
| **Characteristics of patients with CCI** | | | | |
| **Clinical characteristics** | | | | |
| total duration of ICU stay | | τ = .017^1^ | | p = .837 |
| total duration of ventilation | | τ = .069^1^ | | p = .406 |
| Barthel index at admission at post-rehab hospital | | τ = -.006^1^ | | p = .947 |
| Barthel index at discharge from post-rehab hospital | | τ = .044^1^ | | p = .601 |
| Barthel index at discharge from rehab hospital | | τ = -.062^1^ | | p = .460 |
| diagnosis of sepsis | | r = .081^2^ | | p = .466 |
| severity of sepsis | | τ = .007^1^ | | p = .937 |
| Time following ICU discharge | | τ = -.101^1^ | | p = .220 |
| Time following mechanical ventilation | | τ = -.146 | | p = .077 |
| **Socioeconomic characteristics** | | | | |
| age | | τ = -.053^1^ | | p = .524 |
| sex | | r = .084^2^ | | p = .448 |
| family status (living in partnership vs. not living in partnership) | | r = .111^2^ | | p = .317 |
| educational level (≥ 10 years at school vs. < 10 years at school) | | r = -.027^2,3^ | | p = .810 |
| **Acute psychological characteristics** | | | | |
| perceived helplessness at ICU | | τ = -.069^1,3^ | | p = .438 |
| perceived fear of dying at ICU | | r = .007^1,3^ | | p = .943 |
| ASDS^4^ score at ICU | | r = -.209^1,5^ | | **p = .028*** |
| diagnosis of ASD^6^ at ICU (SCID-I ^8^) | | τ = -.211^2,5,7^ | | p = .059 |
| PTSS-10 score (up to six months following ICU) | τ = -.068^1^ | | p = .413 | |
| **Chronic psychological health conditions** | | | | |
| number of traumatic memories from ICU (up to six months following ICU) | | τ = -.137^1,9^ | | p = .133 |
| diagnosis (SCID-I^8^) of PTSD (up to six months following ICU) | | r = -.221^2,9^ | | **p = .046*** |
| previous psychiatric history | | r = -.068^2^ | | p = .540 |
| history of alcohol consumption | | r = -.087^2^ | | p = .433 |
| history of anxiety disorder | | r = -.022^2^ | | p = .844 |
| history of affective disorder | | r = .006^2^ | | p = .960 |
| history of previous traumatic life experiences | | r = -.114^2,10^ | | p = .351 |
| **Characteristics of close family members** | | | | |
| **Socioeconomic characteristics** | |  | |  |
| age | | τ = -.071^1^ | | p = .390 |
| sex | r = -.089^2^ | | p = .422 | |
| **Acute psychological characteristics** | | | | |
| PTSS-10 score (up to six months following ICU) | τ = -.367^1^ | | **p < .001***** | |
| **Chronic psychological health conditions** | | | | |
| history of previous traumatic life experiences | r = -.047^2^ | | p = .674 | |
| **Characteristics of relationship** | | | | |
| perceived satisfaction with relationship | | τ = .101^1,9^ | | p = .274 |
| perceived closeness in relationship | | τ = -.018^1^ | | p = .850 |

^1^ Kendall´s τ; ^2^point-biserial correlation; ^3^n = 3 missing values; ^4^ASDS (Acute Stress Disorder Scale, Helfricht et al., 2009); ^5^n = 2 missing values; ^6^ASD (Acute Stress Disorder); ^7^For parsimony of the final model and because of high correlation between ASDS score and diagnosis of Acute Stress Disorder at ICU (r = .838, p < .001), only ASDS score was included as predictor in multivariate regression model.^8^SCID (Structured Clinical Interview DSM-IV, Wittchen et al., 1997); ^9^n = 1 missing value; ^10^n = 14 missing values; *p<.05; *** p<.001
