# Supplementary material for: Predictors of posttraumatic stress and quality of life in family members of chronically critically ill patients after intensive care
Source: Ann Intensive Care. 2016 Jul 20;6:69. doi: 10.1186/s13613-016-0174-0 (PMC4954797; doi:10.1186/s13613-016-0174-0)
Supplement: Supplementary file 3 — 10.1186/s13613-016-0174-0 Description of univariate correlations. [file 13613_2016_174_MOESM3_ESM.docx]

**Additional file 3: Description of univariate correlations**

*Univariate associations between patient-/ family-related factors and posttraumatic stress in family members*

Regarding patients´ clinical factors, a longer time following ICU discharge and a longer time following mechanical ventilation were positively correlated with posttraumatic stress in family members. With respect to patients´ chronic psychological health conditions, a diagnosis of PTSD and a higher PTSS-10 score three to six months following ICU discharge were positively correlated with posttraumatic distress in family members. A significantly negative correlation between the perceived satisfaction with the relationship and posttraumatic stress in family members could be shown (see Additional file 1: Table S1).

*Univariate associations between patient-/ family-related factors and HRQL in family members*

Regarding patients´ acute psychological characteristic, more severe symptoms of acute stress disorder was negatively correlated with the HRQL in family members. As patients´ chronic psychological health conditions a diagnosis of PTSD was negatively correlated with HRQL in family members. Regarding family members´ acute psychological characteristic, their own posttraumatic stress was significantly negative correlated with HRQL three to six months following ICU discharge (see Additional file 2: Table S2).
