# Supplementary material for: Predictors of posttraumatic stress and quality of life in family members of chronically critically ill patients after intensive care
Source: Ann Intensive Care. 2016 Jul 20;6:69. doi: 10.1186/s13613-016-0174-0 (PMC4954797; doi:10.1186/s13613-016-0174-0)
Supplement: Supplementary file 4 — 10.1186/s13613-016-0174-0 Socio-demographic and clinical characteristics of the patients being followed up (n = 83) and drop outs (n = 112). [file 13613_2016_174_MOESM4_ESM.docx]

**Table S3:** Socio-demographic and clinical characteristics of the patients being followed-up (n = 83) and drop outs (n = 112).

| **Characteristic** | **Patients followed-up**  **n = 83** | **Drop Outs**  **n = 112** | **U/ χ²** | ***p*** |
| --- | --- | --- | --- | --- |
| age, yrs median (IQR) | 61.4 (55.6-65.5) | 61.4 (56.2-66.4) | 4507.000 | .717 (U)^a^ |
| Gender, n (%) |  |  |  |  |
| Male | 61 (73.5) | 82 (73.2) |  |  |
| female | 22 (26.5) | 30 (26.8) | .002 | .965 (χ²)^b^ |
| Family status, n (%) |  |  |  |  |
| Single | 6 (7.2) | 15 (13.4) |  |  |
| Married/cohabited | 63 (75.9) | 71 (63.4) |  |  |
| Divorced/ living apart | 8 (9.6) | 20 (17.9) |  |  |
| widowed | 6 (7.2) | 6 (5.4) | 5.282 | .152 (χ²)^b^ |
| Education, n (%)^c^ |  |  |  |  |
| < 10 yrs | 26 (31.3) | 46 (41.1) |  |  |
| ≥ 10 yrs | 54 (65.1) | 59 (52.7) | 2.443 | .118 (χ²)^b^ |
| ICU stay, days median (IQR) | 66.0 (49.0-97.0) | 71.0 (50.3-103.5) | 4407.500 | .537 (U) |
| Mechanical ventilation , days median (IQR) | 49.0 (33.0-76.0) | 49.5 (32.0-73.0) | 4488.500 | .682 (U) |
| Sepsis, n (%) |  |  |  |  |
| No sepsis | 29 (34.9) | 31 (27.7) |  |  |
| sepsis | 28 (33.7) | 45 (40.2) |  |  |
| Severe sepsis or septic shock | 26 (31.3) | 36 (32.1) | 1.356 | .508 (χ²)^b^ |
| Site of infection, n (%) |  |  |  |  |
| Respiratory | 37 (44.6) | 67 (59.8) | 4.450 | **.035*** (χ²)^b^ |
| Urinary/ genitals | 8 (9.6) | 11 (9.8) | .002 | .966 (χ²)^b^ |
| Abdominal | 8 (9.6) | 7 (6.3) | .771 | .380 (χ²)^b^ |
| Bones/ soft tissue | 5 (6.0) | 5 (4.5) | .238 | .746 (†)^d^ |
| Wound infection | 2 (2.4) | 1 (0.9) | .724 | .576 (†)^d^ |
| Heart | 1 (1.2) | 2 (1.8) | .106 | 1.000 (†)^d^ |
| Multiple | 9 (10.8) | 14 (12.5) | .126 | .723 (χ²)^b^ |
| Others^e^ | 4 (4.8) | 12 (10.7) | 2.200 | .188 (†)^d^ |
| Unknown | 4 (4.8) | 4 (3.6) | .189 | .725 (†)^d^ |
| Barthel index, median (IQR) |  |  |  |  |
| at admission at post-acute ICU | -200.0 (-225.0--140.0) | -185.0 (-225.0- -125.0) | 4390.000 | .502 (U) |
| at discharge from post-acute rehabilitation^f^ | -25.0 (-80.0-10.0) | -45.0 (-115.0- 0.0) | 3755.000 | **.028*(**U) |
| at discharge from rehab hospital | 70.0 (45.0-85.0) | 40.0 (-40.0-75.0) | 3157.000 | **< .001***(**U) |

^a^*p*-value from Mann-Whitney U-Test;^b^*p*-value from χ²-test; ^c^ patients followed up: n = 3 missing values; patients dropped out: n = 7 missing values; ^d^*p*-value from Fisher´s exact test; ^e^patients followed up: n = 1 brain, n = 3 central venous catheter; patients dropped out: n = 8 central venous catheter, n = 1 intracardiac catheter, n = 1 nose, n = 1 portsystem, n = 1 aorta ^f^patients dropped out: n = 1 missing value
