# Supplementary material for: Predictors of posttraumatic stress and quality of life in family members of chronically critically ill patients after intensive care
Source: Ann Intensive Care. 2016 Jul 20;6:69. doi: 10.1186/s13613-016-0174-0 (PMC4954797; doi:10.1186/s13613-016-0174-0)
Supplement: Supplementary file 5 — 10.1186/s13613-016-0174-0 Multiple stepwise regression analysis with patient and family member characteristics as regressors and health-related quality of life in family member of CCI patients as dependent variable N = (83). [file 13613_2016_174_MOESM5_ESM.docx]

Table S4: Multiple stepwise regression analysis with patient and family-member characteristics as regressors and health-related quality of life in family member of CCI patients as dependent variable N = (83).

| **Predictors of health-related quality of life in family members of CCI patients** | **β** | **95% CI** | **t** | **p** |
| --- | --- | --- | --- | --- |
| **Characteristics of patients with CCI** | | | | |
| ASDS score at post-rehab ICU | -.290 | -.360 - -.088 | -3.291 | **.002**** |
| **Characteristics of close family-members** | | | | |
| PTSS score of family members, 3 to 6 months following ICU | -.622 | -.640 - -.358 | -7.064 | **.001***** |
